# Exploring the relationship between functional limitations of the older adults and the health-related quality of life of their spouse in Shaanxi Province, China

**DOI:** 10.1186/s12955-021-01835-4

**Published:** 2021-08-30

**Authors:** Wanyue Dong, Anthony B. Zwi, Chi Shen, Yue Wu, Jianmin Gao

**Affiliations:** 1 School of Health Economics and Management, Nanjing University of Chinese Medicine, Nanjing, China; 2 School of Social Sciences, The University of New South Wales, Sydney, Australia; 3 School of Public Policy and Administration, Xi’an Jiaotong University, Xi’an, China

**Keywords:** Health-related quality of life, Functional limitations, Spousal health, Disability, Aging

## Abstract

**Background:**

With trends towards longer life expectancy, lifetime with disability has also been prolonged. It is increasingly recognized that not only the person with disability but also those around them are affected. The relationship between functional limitation (FL) of the older adults and health-related quality of life (HRQoL) of their spouse is of interest. So too is the determination of the factors aside from FL that influence HRQoL.

**Methods:**

The sample was derived from the 2013 National Health Service Survey conducted in Shaanxi Province in China. Married couples aged ≥ 60 years were selected (n = 3463). The European quality of life five dimensions (EQ-5D) and visual analogue scale were used to measure HRQoL.

**Results:**

Both wife and husband reported lower HRQoL if either the male or female partner had some or serious FLs (*P* < 0.001). Other factors associated with lower HRQoL of the spouse included age, lower educational level, presence of chronic disease, and lower household economic status. Family size was associated with wife's HRQoL only when the male had no FL and lived with another 1–2 persons, or when the male had some FLs and lived in a larger family (n ≥ 5). Residential status did not relate to the HRQoL of spouses regardless of FL status.

**Conclusions:**

Older adults in Shaanxi province who have partners with FLs tend to report poorer EQ-5D, suggesting that couples amongst whom one has FL may be particularly vulnerable to lower HRQoL.

## Background

Ill health affects more than the individual suffering from an acute or chronic condition; it also may influence (directly or indirectly) the physical and mental health, quality of life, and life satisfaction of a broader social network around an affected individual [1–4]. With recent trends towards more extended life expectancy, lifetime lived with disability is also prolonged, and this may have a ‘spillover’ effect on the health status of surrounding individuals, especially spouses. The nature and influences of such relationships require further examination [5].

There is growing consensus that the impact of ill-health on family members needs to be considered in economic evaluation [4, 6, 7]. However, even where this is undertaken, most analyses typically include direct costs associated with caregiving, while the possible decline in health-related quality of life (HRQoL) of family members is implicitly assumed to be zero [8]. Understanding the relationship between functional limitations (FL) of older people, and the HRQoL of their spouses, may be essential for health technology assessment and considering interventions to support older adults and their families.

Previous studies have documented associations between specific diseases in older adults (e.g. Alzheimer’s disease, dementia, cancer, meningitis, and stroke) and the burden on caregivers both in terms of health effects and quality of life, but generalizability to the entire elder population was limited [9–13]. Existing measurement instruments are generally limited to the caregiver her/himself, and the impact on and experience of specific members of the family may not be appreciated [7]. Elder spouses may suffer additional burdens due to experiences such as irregular employment, little choice over care-giving roles given shared living space, and high-intensity care needs compared to their own, resulting from aging and related morbidities [5, 14–16]. Their burden increases significantly as functional and cognitive impairments are imposed by limiting the patient’s ability to care for himself [17]. Exploring whether FL is associated with declines in HRQoL in spouses is important, given a common preference among older people to remain in their local community and maintain their social networks throughout the aging process [18].

China has the most significant number of older adults over 60 years old (249.5 million by the end of 2018) in the world, and this proportion (17.9%) is expected to be over 30% by 2050 [19, 20]. In the next few years, the number of older adults with FL will also increase dramatically [21]. However, most of the relevant research has been conducted in countries with advanced economies, and few studies have examined whether the correlations observed elsewhere are replicated in China's aging population [21]. It is essential to determine whether the relationship between FL of the older adults and HRQoL of their spouses is confirmed in the context of China, given the critical role of informal care in the elderly and the traditional close-knit family structure in China.

This study focused on secondary analysis of Chinese population data and sought to analyze the relationship between FL of the older adults and HRQoL of their spouses, and explore what other factors influenced this relationship.

## Methods

### Data

**Fig.1:**
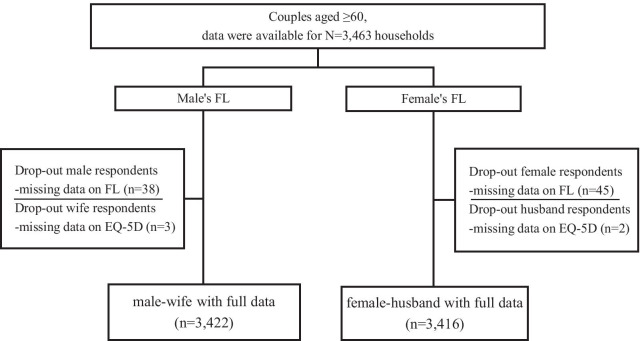
Participants in the study and inclusion at different stages of analysis

The study population was derived from the National Health Service Surveys (NHSS) conducted in Shaanxi Province in 2013. The NHSS is a representative survey using a multi-stage stratified cluster random sampling method. All members in each selected household were interviewed individually using a structured household questionnaire, and a total of 20,700 households were surveyed. The analyses presented here focused on married respondents both aged ≥ 60 years old (n = 3463). The questionnaire is required to be answered by the older adults themselves. If the older adult is not present or unable to answer, the questionnaire will be answered by the family member. To ensure the validity of the answer, the quality control requires that the response rate by adult respondents themselves is not less than 70%. Given possible gender differences in the relation of spouse HRQoL to partner FL, male and female samples were analyzed separately [22]. The model then produced two estimates of interest: the relationship between the male index elder’s FL and his wife’s HRQoL, and the relationship between the female index elder’s FL and her husband's HRQoL. Figure 1 displays the inclusion of spouses in the study and the number of spouses included in each analysis stage. A total of 3422 male's FL and 3416 female's FL were recruited to the study after processing the logical errors and removing incomplete or missing data.

### Measures

HRQoL was assessed using European Quality of Life five dimensions (EQ-5D) and Visual analogue scale (VAS), a generic instrument for measuring health status among older adults [23–25]. The EQ-5D-3L comprises five domains: mobility, self-care, usual activities, pain/discomfort, and anxiety/depression, scoring from 1 (no problems) to 3 (extreme problems) [24–26]. In addition, VAS records the respondent’s self-rated health by asking the older adults to place a mark on the scale of 100 mm line anchored that corresponds to their HRQoL, ranging from 0 (the worst imaginable health) to 100 (the best imaginable health) [27]. These two methods are considered to have good validity and reliability [28].

In our study, FL considers the limitations of daily activities presenting for 6 months or more, and incorporates three items concerning mobility, auditory, and visual elements, each assessed as having “no problems”, “some problems” or “serious problems”. FL of the older adults, was then grouped into three categories depending on the above: “No FL” if there were no problems in mobility, auditory, or visual dimensions in any area; “Some FLs” where some problems among mobility, auditory, or visual function were detected, but none were severe; and “Serious FLs” where at least one problem rated as serious was present concerning mobility, auditory, or visual functions.

The demographic characteristics of the older adults and their spouses were included as explanatory variables. Covariates of the older adults and spouses included age, educational level, and presence of chronic conditions. Residential status, family size, and household economic level were included as household characteristics. The age of the older adults and spouses was entered as continuous variables. Educational level was divided into illiterate, elementary, middle school, or high school and above. Chronic conditions were classified as present or absent due to differences in response categories. Residential status was categorized as urban or rural with family size into three groups (2 people, 3–4 people, or ≥ 5 people). The nett household expenditure (total household expenditure in the last year minus household health expenditure) was measured to reflect household economic level as a continuous variable in this study [29].

### Statistical analyses

Descriptive statistics were presented as mean (standard deviation) or frequency (percentage). The distribution of EQ-5D dimensions of spouses among different groups was compared using χ^2^ test. For comparing the median VAS value, the Kruskal–Wallis test was used.

The internal consistency reliability was measured using Cronbach’s alpha for the EQ-5D and FL. The results were 0.863, and 0.784, respectively. The exploratory factor analysis (EFA) was appropriate as indicated by a Kaiser–Meyer–Olkin (KMO) measure of sampling adequacy of 0.846 and a highly significant Bartlett’s Test of Sphericity (*p* < 0.001), showing two common factors with a cumulative contribution rate of 59.61% extracted.

To investigate the relationship between the elder’s FL and his/her spouse’s HRQoL, a 2-level mixed linear model was adopted with the log of spouse’s VAS value as the dependent variable. The following characteristics were considered as independent variables: (1) Individual level: Elderly-related variables including FL, age, educational level and chronic conditions; Spouse’s-related variables including age, educational level and chronic conditions; and (2) Household level: residential status, family size, and household economic level [30]. Stratified analyses investigated the influence of family size and residential status on the HRQoL within different FL groups. All analyses were conducted using STATA version 14.0.

## Results

The FL categories are tabulated in Table 1. A total of 3422 and 3416 couples were analyzed respectively based on gender. According to the male index elders’ health status, 1800, 1160, and 462 couples were grouped into “no FL”, “some FLs” and “serious FLs” respectively, compared to 1899, 1076, and 441 couples based on female index elders’ health status.

**Table 1:** Overall of the groups description (n)

	Male index elders’ health status			Female index elders’ health status		
	(n = 3422)			(n = 3416)		
	No FL	Some FLs	Serious FLs	No FL	Some FLs	Serious FLs
Mobility	1800	155	170	1899	202	193
Auditory	1800	773	242	1899	586	193
Visual	1800	690	121	1899	722	126
Total	1800	1160	462	1899	1076	441

Characteristics of couples in different FL groups are shown in Table 2. Males were generally about 3 years older, and had higher educational level than females. Over half of the respondents were illiterate or had only completed elementary school. Nearly three-quarters (73.88%) of respondents lived in rural areas, and about half (42.42%) lived with at least one additional family member aside from their spouse. The proportion of respondents with chronic diseases were higher in the “some FLs” and “serious FLs” groups than the “no FL” group.

**Table 2:** Characteristics of respondents in different FL groups [${\overline{\text{x}}}$ ± s/n (%)]

Variables	Male index elders and their wives						Female index elders and their husbands					
	No FL		Some FLs		Serious FLs		No FL		Some FLs		Serious FLs	
	(n = 1800)		(n = 1160)		(n = 462)		(n = 1899)		(n = 1076)		(n = 441)	
	Male	Wife	Male	Wife	Male	Wife	Female	Husband	Female	Husband	Female	Husband
Age (years)	68.16 ± 5.59	65.72 ± 4.96	70.92 ± 6.29	67.96 ± 5.74	72.60 ± 7.31	69.31 ± 6.42	65.81 ± 5.00	68.48 ± 5.86	68.11 ± 5.83	70.93 ± 6.45	69.17 ± 6.38	71.96 ± 6.76
Education												
Illiteracy	268	631	269	536	117	231	673	307	508	248	215	99
	(14.89)	(35.06)	(23.19)	(46.21)	(25.32)	(50.00)	(35.44)	(16.17)	(47.21)	(23.05)	(48.75)	(22.45)
Elementary	658	676	438	391	170	158	716	692	363	408	147	163
	(36.56)	(37.56)	(37.76)	(33.71)	(36.80)	(34.20)	(37.70)	(36.44)	(33.74)	(37.92)	(33.33)	(36.96)
Middle school	560	343	289	158	114	52	364	575	133	269	52	115
	(31.11)	(19.06)	(24.91)	(13.62)	(24.68)	(11.26)	(19.17)	(30.28)	(12.36)	(25.00)	(11.79)	(26.08)
High school and above	314	150	164	75	61	21	146	325	72	151	27	64
	(17.44)	(8.32)	(14.14)	(6.46)	(13.20)	(4.54)	(7.69)	(17.11)	(6.69)	(14.03)	(6.13)	(14.51)
Chronic disease												
No	1,093	977	558	537	230	219	1,090	1,078	470	568	171	231
	(60.72)	(54.28)	(48.10)	(46.29)	(49.78)	(47.40)	(57.40)	(56.77)	(43.68)	(52.79)	(38.78)	(52.38)
Yes	707	823	602	623	232	243	809	821	606	508	270	210
	(39.28)	(45.72)	(51.90)	(53.71)	(50.22)	(52.60)	(42.60)	(43.23)	(56.32)	(47.21)	(61.22)	(47.62)
Residential												
Rural	1,260	1,260	835	835	352	352	1,336	1,336	775	775	329	329
	(70.00)	(70.00)	(71.98)	(71.98)	(76.19)	(76.19)	(70.35)	(70.35)	(72.03)	(72.03)	(74.60)	(74.60)
Urban	540	540	325	325	110	110	563	563	301	301	112	112
	(30.00)	(30.00)	(28.02)	(28.02)	(23.81)	(23.81)	(29.65)	(29.65)	(27.97)	(27.97)	(25.40)	(25.40)
Family size												
2	1,019	1,019	698	698	254	254	1,082	1,082	654	654	232	232
	(56.61)	(56.61)	(60.17)	(60.17)	(54.98)	(54.98)	(56.98)	(56.98)	(60.78)	(60.78)	(52.61)	(52.61)
3–4	435	435	270	270	112	112	451	451	250	250	114	114
	(24.17)	(24.17)	(23.28)	(23.28)	(24.24)	(24.24)	(23.75)	(23.75)	(23.23)	(23.23)	(25.85)	(25.85)
≥ 5	346	346	192	192	96	96	366	366	172	172	95	95
	(19.22)	(19.22)	(16.55)	(16.55)	(20.78)	(20.78)	(19.27)	(19.27)	(15.99)	(15.99)	(21.54)	(21.54)
Economic status (Yuan)	6580.34 ± 5066.45	6580.34 ± 5066.45	5862.72 ± 4866.58	5862.72 ± 4866.58	5521.35 ± 4496.83	5521.35 ± 4496.83	6495.5 ± 5084.62	6495.5 ± 5084.62	5861.78 ± 4761.38	5861.78 ± 4761.38	5777.75 ± 4713.81	5777.75 ± 4713.81

Table 3 shows the answers to the EQ-5D reported by the spouses to the index elders’ FL category. Both wife and husband reported lower HRQoL in all dimensions of EQ-5D if male or female index elders had “some FLs” or “serious FLs” and the differences are statistically significant (*P* < 0.001). This means that for a male index elder reporting some or serious FLs, his wife tends to have a higher probability of having one or more problems to mobility, self-care, usual activities, pain/discomfort, or anxiety/depression. The same type of relationship was also found in the VAS scores.

**Table 3:** Spouses-reported answers to EQ-5D and VAS in response to the index elders' FL by dimensions and groups [n (%)/median (IQ)]

Spouses' EQ-5D to elders	Functional limitation category							
	Male index elders			*p* value	Female index elders			*p* value
	No FL	Some FLs	Serious FLs		No FL	Some FLs	Serious FLs	
	(n = 1800)	(n = 1160)	(n = 462)		(n = 1899)	(n = 1076)	(n = 441)	
Mobility				< 0.001				< 0.001
No problem	1588	874	339		1678	820	331	
	(88.22)	(75.35)	(73.38)		(88.36)	(76.21)	(75.06)	
Some problems	191	263	104		200	241	101	
	(10.61)	(22.67)	(22.51)		(10.53)	(22.40)	(22.90)	
Serious problems	21	23	19		21	15	9	
	(1.17)	(1.98)	(4.11)		(1.11)	(1.39)	(2.04)	
Self-care				< 0.001				< 0.001
No problem	1679	1005	374		1782	938	373	
	(93.28)	(86.64)	(80.95)		(93.84)	(87.17)	(84.58)	
Some problems	99	127	62		95	114	59	
	(5.50)	(10.95)	(13.42)		(5.00)	(10.59)	(13.38)	
Serious problems	22	28	26		22	24	9	
	(1.22)	(2.41)	(5.63)		(1.16)	(2.24)	(2.04)	
Usual activities				< 0.001				< 0.001
No problem	1624	908	345		1714	858	341	
	(90.22)	(78.28)	(74.68)		(90.26)	(79.74)	(77.32)	
Some problems	140	207	82		151	180	78	
	(7.78)	(17.84)	(17.75)		(7.95)	(16.73)	(17.69)	
Serious problems	36	45	35		34	38	22	
	(2.00)	(3.88)	(7.57)		(1.79)	(3.53)	(4.99)	
Pain/discomfort				< 0.001				< 0.001
No problem	1361	698	274		1489	694	294	
	(75.61)	(60.17)	(59.31)		(78.41)	(64.50)	(66.67)	
Some problems	419	423	163		385	364	133	
	(23.28)	(36.47)	(35.28)		(20.27)	(33.83)	(30.16)	
Serious problems	20	39	25		25	18	14	
	(1.11)	(3.36)	(5.41)		(1.32)	(1.67)	(3.17)	
Anxiety/depression				< 0.001				< 0.001
No problem	1629	926	353		1737	909	361	
	(90.50)	(79.83)	(76.41)		(91.47)	(84.48)	(81.86)	
Some problems	159	217	98		155	161	73	
	(8.83)	(18.71)	(21.21)		(8.16)	(14.96)	(16.55)	
Serious problems	12	17	11		7	6	7	
	(0.67)	(1.46)	(2.38)		(0.37)	(0.56)	(1.59)	
VAS	80	70	70	< 0.001	80	70	70	< 0.001
Median (IQ range)	(70–81)	(60–80)	(60–80)		(70–85)	(60–80)	(60–80)	

**Fig. 2:**
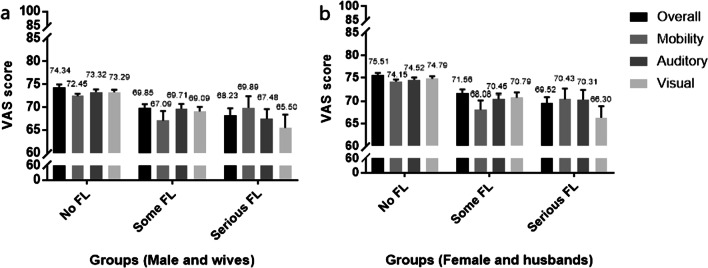
Spouses’ VAS score with 95% confidence interval in response to the index elders' FL by dimensions and groups

As shown in Fig. 2, for those elders (male and female) who had some or serious FLs, their wife/husband had a lower VAS score than those without FL. Table 4 demonstrates that these differences are statistically significant (all *P* < 0.001). Own aging, lower educational level, presence of chronic disease, and lower household economic status were all associated with a lower reported VAS score of the spouses. It is worth noting that in addition to the above self-factors, female index elders’ educational level and chronic disease also had an impact on husbands’ VAS score, as shown in Table 4.

**Table 4:** Regression results for log (VAS) distribution of spouse in relation to the index elder within different FL groups

Variables	Male index elders and wives			Female index elders and husbands		
	Coef	SD	95% CI	Coef	SD	95% CI
Group						
Some FLs	− 0.041**	0.009	(− 0.058 to − 0.023)	− 0.035**	0.010	(− 0.054 to − 0.016)
Serious FLs	− 0.080**	0.019	(− 0.118 to − 0.043)	− 0.057**	0.013	(− 0.083 to − 0.032)
Wife age	− 0.006**	0.001	(− 0.009 to − 0.003)	− 0.001	0.001	(− 0.004 to 0.002)
Wife education						
Elementary	0.033**	0.011	(0.013–0.054)	0.027*	0.011	(0.006–0.048)
Middle school	0.059**	0.015	(0.031–0.088)	0.044**	0.013	(0.019–0.069)
High school and above	0.059*^a^	0.021	(0.018–0.099)	0.029	0.017	(-0.003–0.062)
Wife chronic disease						
Yes	− 0.109**	0.009	(− 0.126 to − 0.091)	− 0.015	0.009	(− 0.032 to − 0.002)
Husband age	0.001	0.001	(− 0.001 to 0.004)	− 0.004**	0.001	(− 0.006 to − 0.001)
Husband education						
Elementary	0.004	0.014	(− 0.022 to 0.031)	− 0.008	0.012	(− 0.033 to 0.016)
Middle school	− 0.011	0.015	(− 0.041 to 0.018)	0.011	0.014	(− 0.015 to 0.038)
High school and above	0.002	0.021	(− 0.038 to 0.043)	0.040*	0.017	(0.007–0.072)
Husband chronic disease						
Yes	− 0.006	0.009	(− 0.023 to 0.011)	− 0.102**	0.008	(− 0.118 to − 0.085)
Residential						
Urban	− 0.012	0.014	(− 0.040 to 0.016)	− 0.013	0.014	(− 0.041 to 0.015)
Family size						
3–4	− 0.016	0.010	(− 0.036 to 0.004)	− 0.018	0.011	(− 0.039 to 0.003)
≥ 5	− 0.003	0.014	(− 0.031 to 0.025)	0.011	0.01	(− 0.007 to 0.031)
Economic status	0.020**	0.006	(0.008–0.032)	0.026**	0.008	(0.011–0.042)
_cons	4.487	0.079	(4.333–4.642)	4.435	0.097	(4.245–4.624)

^a^* and ** denote statistical significance at 5 and 1% level, respectively

Both family size and residential status were essential factors concerning HRQoL in addition to FL. To further analyze this, we controlled other social characteristics to see the effect of these factors. Tables 5 and 6 show that if male index elders had no FL, and the wives living with another 1–2 persons aside from the spouse, the wives exhibited lower VAS scores than couples living alone. If the male index elders had some FLs and lived together in a bigger family (n ≥ 5), then the wives had higher VAS scores than couples living alone. The same situation was not observed in the case of husbands in relation to their female index elders' FL status. In all FL groups, residential status was not associated with the spouse’s VAS score.

**Table 5:** Regression results of log (VAS) distribution of wives in relation to the male index elders' FL by family size and residential categories

Spouses’ log(VAS) in relation to elders	Male functional limitation category					
	No FL		Some FLs		Serious FLs	
	(n = 1800)		(n = 1160)		(n = 462)	
	Coef	95% CI	Coef	95% CI	Coef	95% CI
Family size^a^						
3–4	− 0.037**^b^	(− 0.063 to − 0.012)	0.012	(− 0.023 to 0.048)	0.016	(− 0.050 to 0.082)
≥ 5	− 0.010	(− 0.036 to 0.016)	0.039*	(0.001–0.076)	− 0.054	(− 0.189 to 0.082)
Residential status						
Urban	− 0.022	(− 0.056 to 0.011)	0.001	(− 0.040 to 0.041)	0.018	(− 0.104 to 0.140)

^a^All specific include controls for age, education, chronic disease, and household economic level

^b^* and ** denote statistical significance at 5 and 1% level, respectively

**Table 6:** Regression results of log (VAS) distribution of husbands in relation to the female index elders’ FL by family size and residential categories

Spouses’ log(VAS) in relation to elders	Female functional limitation category					
	No FL		Some FLs		Serious FLs	
	(n = 1899)		(n = 1076)		(n = 441)	
	Coef	95% CI	Coef	95% CI	Coef	95% CI
Family size^a^						
3–4	− 0.024	(− 0.053 to 0.005)	− 0.011	(− 0.048 to 0.025)	− 0.011	(− 0.069 to 0.048)
≥ 5	0.004	(− 0.019 to 0.028)	0.027	(− 0.014 to 0.069)	0.009	(− 0.044 to 0.062)
Residential status						
Urban	− 0.010	(− 0.040 to 0.019)	− 0.027	(− 0.089 to 0.034)	0.016	(− 0.068 to 0.099)

^a^All specific include controls for age, education, chronic disease, and household economic level

## Discussion

This cross-sectional study reports on an analysis of Chinese population data, focusing on examining the link between FL of those aged 60 or more years, and their spouse’s HRQoL. The results could help inform health care decisions as well as helping to determine the health costs and benefits associated with older couples and their families, and potentially involving multiple individuals in health interventions [6, 31].The results indicated that the spouse of an older adult with mild or more severe FLs tended to report poorer EQ-5D, both for males and females. Some researchers attribute these links to the ‘spillovers’ of illness or disability of an elder on the other family members. The HRQoL of partners is often affected by the elder FL among couples since they often share emotional distress, with a more frequent and more substantial need for emotional and physical support [32]. Consistent with much research, our study adds empirical, population-level evidence to the literature demonstrating the existence of 'spillovers' from functional limitations onto the quality of life and health of partners [5, 9].

For both male and female elders, we found that spouses’ HRQoL was related to spouses’ age, lower educational level, presenting chronic disease, as well as lower household economic level. In addition, for only female elders' lower educational level and showing chronic condition was associated with lower HRQoL of their husbands. This difference may relate to the perspective that women express emotions more freely than men [33]. However, whether the negative sentiment from elder women impacts their husbands’ HRQoL in the family remains to be explored.

In our study, family size played an essential role in relation to the wife's HRQoL, but this was not observed in relation to husbands. An explanation for the gender difference may be the unequal support that men and women receive in the family [34]. When males had “no FL”, the wife’s HRQoL was lower among those who lived with another 1–2 persons. One possible explanation may be the increased burden on older women who need to take care of grandchildren left by their adult children who migrate internally as part of the urbanization trend in China, but data on who the elders were living with was not available [35]. When males had “some FLs”, the wife's HRQoL was higher in the presence of a larger family, and may relate to the responsibility of providing care (to elders, their spouses, or younger children) being distributed across the range of family members present.

Our results indicate that older adults in Shaanxi province who have partners with FLs tend to report poorer EQ-5D. From a policy point of view, China advocates home care for older adults, but the focus of care is limited to individuals. At present, there is still a lack of family policies that specifically support and assist spouses of older adults with disabilities. Besides, the social value of home care has not been encouraged because the care services provided by family members are difficult to measure. Therefore, we must start with interventions to provide the most stable and long-term support for these vulnerable groups. The government should provide financial compensation to the older adult families, which can not only relieve the financial pressure of the spouses, but also increase their willingness and ability to provide care enthusiasm for caring. In addition, some employment support policies can also be provided, such as providing flexible working mechanisms for family members, and providing support for the vocational skills training needed to return to the labor market [36]. Policymakers can also learn from the Lifespan Respite Care Act in the US to clarify the role and social identity of family caregivers in the form of laws and regulations [37].

Unlike previous literature focusing on specific diseases, we are concerned with the common features in the older adults. Thus our sample included the older adults living in a household [5]. FL and HRQoL have the most significant importance for older people when limited life expectancy and increasing clinical complexity predominate [34]. Our study included a population-based sample and compared the association of elders with no, some or serious FL on the EQ-5D and VAS of their partners. A separate analysis of male and female elders raised the possibility that differences in the HRQoL of their spouses may relate to gendered differences in roles as well as in physiological and psychological responses, and is consistent with other reports [34]. The dataset allows us to explore the relationship between FL of the older adults and HRQoL of spouses and the social factors aside from FL, such as family size and residential status, that may relate to HRQoL of spouses.

Several potential limitations should be noted. First, the cross-sectional design does not help us determine the cause-and-effect relationship between the FL and HRQoL. In addition, we recognize that the older adults' condition-specific factors, including activities of daily living (ADL), FL duration, the burden of care imposed on the spouse, may affect HRQoL in couples, but were not considered due to limitations in the data available to us. Moreover, the role of family living mode, such as living with grandchildren, on elderly wife's HRQoL needs to be further explored once such data are available available. Furthermore, the differential impact of the FL of the elder in relation to the effect of the partner's health, on partner HRQoL, was not able to be assessed. Thus, further longitudinal studies are needed to establish the causal relationships between FL of the older adults and HRQoL of their spouses. Beyond exploring these crucial relationships, integrating these insights into clinical, therapeutic, economic and evidence-informed policy decision-making remains to be explored.

## Conclusions

We investigated the association between the FL of an older adult in China with the HRQoL of that person's spouse. We found evidence that the partners of those with FLs tended to report poorer EQ-5D, suggesting that couples amongst whom one has FLs may be particularly vulnerable to lower HRQoL. Family size also influences this relationship, but it only affects the wife’s HRQoL in some cases.

## Data Availability

The datasets generated during the current study are available from the corresponding author on request.

## Abbreviations

ADL: Activities of daily living
EFA: Exploratory factor analysis
FL: Functional limitation
HRQoL: Health-related quality of life
KMO: Kaiser–Meyer–Olkin
NHSS: National Health Service Survey
EQ-5D: European quality of life five dimensions
VAS: Visual analogue scale
